# The Effect of Iron Replacement Therapy on HbA1c Levels in Diabetic and Nondiabetic Patients: A Systematic Review and Meta-Analysis

**DOI:** 10.3390/jcm12237287

**Published:** 2023-11-24

**Authors:** Amani M. AlQarni, Amal A. Alghamdi, Hussain J. Aljubran, Omar A. Bamalan, Abdullah H. Abuzaid, Mohammed A. AlYahya

**Affiliations:** Department of Family and Community Medicine, College of Medicine, Imam Abdulrahman Bin Faisal University, Dammam 31441, Saudi Arabia; amlalghamdi@iau.edu.sa (A.A.A.); 2180001511@iau.edu.sa (H.J.A.); 2180001260@iau.edu.sa (O.A.B.); 2180005606@iau.edu.sa (A.H.A.); 2190003361@iau.edu.sa (M.A.A.)

**Keywords:** iron-deficiency anemia, iron replacement therapy, HbA1c, glycated hemoglobin, diabetes

## Abstract

Background: Several studies have reported that iron-deficiency anemia (IDA) and its treatment might lead to a distorted reading of glycated hemoglobin (HbA1c) value. Hence, this review aims to systematically investigate the effect of iron replacement therapy (IRT) on HbA1c levels, as the literature is deficient in assessing this clinical phenomenon. Methods: An electronic search of the Cochrane, MEDLINE, and Embase databases was conducted by four independent authors. Results: Among the 8332 articles identified using the search strategy, 10 records (with a total of 2113 participants) met the inclusion criteria and were analyzed. In nine of the studies, IRT was found to decrease HbA1c levels; in the remaining study, IRT was found to increase HbA1c levels. The effect size of the pooled standardized mean difference in HbA1c levels between the treatment and control groups with IDA was 1.8 (95% CI = −0.5, 2.31). Heterogeneity was assessed using the I^2^ and χ^2^ tests, and the resultant values were 98.46% and *p* = 0.09, respectively. Additionally, the mean difference between the HbA1c levels (pre-IRT and post-IRT) showed a drop in the HbA1c levels which ranged from 1.20 to 0.43 mg/dL. Conclusions: The results suggest that IRT decreases HbA1c levels, and it is helpful in treating IDA patients with poor glycemic control. Accordingly, the results provide an added perspective on antidiabetic medication dosing and physicians’ interpretation of initially elevated HbA1c values.

## 1. Introduction

Hemoglobin is a major component of blood and includes heme, an iron-binding porphyrin. The essentiality of iron in human physiology is evident by its complex multifunctionality. For example, the iron content of pancreatic islet beta cells is intimately connected to blood glucose levels; therefore, iron plays a role in the onset and progression of diabetes mellitus (DM) [1,2,3]. Hence, increasing our understanding of the iron metabolism process helps us to better comprehend the effects of iron deficiency or anemia on an individual’s glycemic status. More specifically, individuals with conditions which affect their ability to metabolize iron may have low iron levels and develop iron-deficiency anemia (IDA), which may subsequently affect their glycemic status, particularly their glycated hemoglobin (HbA1c) levels [4,5,6].

HbA1c is generated when the valine in the hemoglobin beta chain is glycated in the serum, and is the most important component of the HbA1 group in the monitoring of DM. The practice of using the HbA1c level as a diagnostic indicator of the estimated mean blood glycation level over the previous two to three months was approved and introduced in the 1980s [7,8]. More recently, the American Diabetes Association (ADA) has approved the assessment of HbA1c levels as a gold standard diagnostic test for DM and a follow-up tool for assessing glycemic control; hence, it has become a keystone of clinical practice [9,10]. Although testing for HbA1c is more practical than conducting other tests in clinical settings, there are several circumstances which can affect the accuracy of its outcome [7,8,9].

IDA is one of the most important factors affecting HbA1c levels. In 1965, Horton and Huisman published the first study on the correlation between IDA and HbA1c levels, and several other studies have since been published on the influence of IDA on HbA1c levels [11]. While most of these studies found that IDA led to falsely elevated HbA1c levels [12,13,14,15,16,17], others found that it resulted in falsely reduced HbA1c levels [18,19,20,21,22] or had no effect on HbA1c levels [23,24]. Furthermore, several studies found that the severity of IDA is directly proportional to its effect on HbA1c levels [25,26,27,28]. Therefore, caution should be taken when diagnosing prediabetes or DM in patients with IDA and HbA1c levels which are close to the diagnostic threshold for DM.

Appropriate and timely management of IDA is vital to improve the patient’s quality of life, reduce the need for blood transfusions, and alleviate symptoms. Both oral and intravenous iron replacement therapy (IRT) are treatment options; however, oral IRT is ineffective for treating some diseases (e.g., malabsorptive gastrointestinal diseases) [29]. The correlation between IDA and HbA1c levels has been extensively studied in patients with IDA before and after IRT, and while most of the studies found that IRT led to a reduction in HbA1c [30,31,32,33,34,35,36], some studies found increases in HbA1c levels after IRT [19,37]. Nevertheless, these studies were conducted with relatively small samples, and the IRT protocols varied among the studies. Thus, this study aims to elucidate the impact of IRT on HbA1c levels and provide practical recommendations for managing individuals with IDA and inadequate glycemic control in clinical practice.

## 2. Materials and Methods

### 2.1. Protocol and Registration

All researchers involved in this study followed the Cochrane Review methods and the Preferred Reporting Items for Systematic Reviews and Meta-Analyses (PRISMA) guidelines. In addition, the study was registered on Prospero (registration no. CRD42022312203) [38].

### 2.2. Eligibility Criteria

Articles which described studies conducted with humans of any age, gender, or race with an IDA diagnosis were included. For inclusion, the described studies needed to include pre- and postintervention data on iron and HbA1c parameters from IDA patients treated with IRT. Studies with the following types of study design were eligible for inclusion: case—control, cross-sectional, cohort, and randomized controlled trials, published in any language.

When any of the following criteria were met, the articles were excluded from the study: duplicated clinical data published in different periodicals, poor quality manuscripts, nonprimary studies, inclusion of participants with coexisting non-iron-deficiency anemias, no use of HbA1c as an indicator of DM, or no IDA measurement included.

### 2.3. Information Sources

The electronic search for eligible studies was conducted using the Cochrane Central Register of Controlled Trials (OvidSP), MEDLINE (ProQuest, Ann Arbor, MI, USA), and Embase (OvidSP) databases. In each case, the publication date range was set as January 1956 to October 2022. Furthermore, we surveyed the reference lists of all eligible records and searched Google Scholar to identify any additional eligible records.

### 2.4. Search Strategy

Two main keyword groups were used in the search strategy. One group included all words related to IDA, while the other group included all words related to HbA1c. The full list of the keywords used is provided in Supplementary Table S1. These search terms were determined by inspecting the titles and abstracts, subject indexing relevant studies, and using the PubMed PubReMiner word frequency analysis tool.

### 2.5. Selection Process

The search process involved importing articles and reference lists into Mendeley, where duplicates were identified, reviewed manually, and eliminated. Subsequently, a three-step filtering process was employed, which entailed evaluating the records based on their title, abstract, and full text. The records were divided into two groups. One group was independently evaluated by O.A.B. and M.A.Y., while the other was evaluated by H.J.J and A.H.A. In cases of disagreement, consensus was reached by discussion, and if necessary, a fifth researcher (A.A.G) was consulted. Non-English records were translated using Google Translate to determine their eligibility. Further information was sought from the authors of seven studies to clarify necessary details and acquire the needed data.

### 2.6. Data Collection Process

The designed data extraction form included the following fields: (1) study identifiers and characteristics of the study design (i.e., authors, country, study design, sample size, median age of participants, and percentage of female participants); (2) characteristics of the exposure and comparator groups related to IDA before and after IRT (i.e., hemoglobin, ferritin, transferrin, iron level, total iron-binding capacity [TIBC], mean corpuscular volume [MCV], mean corpuscular hemoglobin [MCH], mean corpuscular hemoglobin concentration [MCHC], red blood cell [RBC] count, hematocrit, and red blood cell distribution width [RDW]); and (3) characteristics of the exposure and comparator groups related to DM and HbA1c before and after IRT (i.e., number of DM patients in case groups, number of DM patients in control groups, and methods used to measure HbA1c levels and postprandial blood sugar). The form was pilot tested by all study team members using three known relevant records, and after confirming its functionality, the reviewers worked independently to extract the data from the selected records.

### 2.7. Study Risk of Bias Assessment

To assess the quality of the included studies, Joanna Briggs Institute critical appraisal checklists were utilized [39,40]. These checklists are used to evaluate the appropriate bias category (information bias, selection bias, confounding) and the quality of the study design and the statistical analysis. Then, based on the risk of bias, each study was classified as low, intermediate, or high risk. Further details of the assessment of bias are provided in Supplementary Tables S2 and S3.

### 2.8. Statistical Analysis

STATA version 18 was used to calculate the Hedge g standardized mean difference in the HbA1c levels between the treatment and control groups, using a random effect model with restricted maximum likelihood (REML) estimation. A forest plot was generated to visually represent the distribution of the mean difference in the HbA1c levels before and after treatment with IRT and the standardized mean difference (SMD) meta-analysis results. The presence and magnitude of any heterogeneity were assessed using the χ^2^ and I^2^ tests and a Galbraith graph. A funnel plot was generated to evaluate the presence of publication bias.

## 3. Results

### 3.1. Study Selection

**Figure 1:**
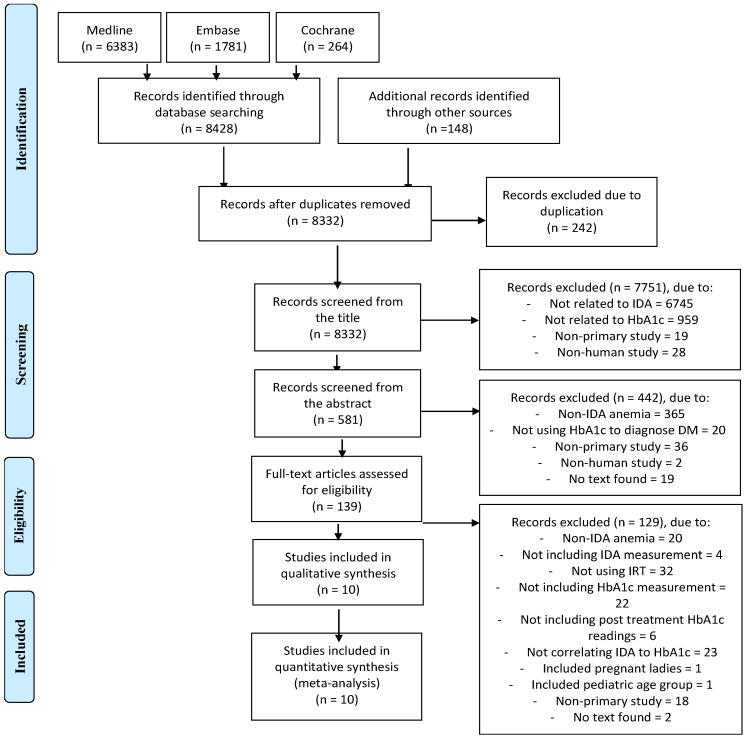
PRISMA flow diagram of the search process.

A thorough search of the literature resulted in the collection of 8332 unique articles (Supplementary Table S4). After reviewing the titles and abstracts, 139 studies were found which met the eligibility criteria. Among these 139 studies, only 10 were ultimately deemed suitable for our purposes and included in the study, while the others were excluded for the reasons outlined in the PRISMA diagram shown in Figure 1.

### 3.2. Bias Assessment

In Supplementary Table S5, a detailed evaluation of the appropriate bias category (information bias, selection bias, confounding) and the quality of the statistical analysis of each study is provided. Among the 10 included studies, seven were found to have a low risk of information bias, and three studies were found to have an intermediate risk. Regarding selection bias, four studies were labeled as low risk, four as intermediate risk, and two as high risk. In terms of confounding, three studies were found to have a low risk, four studies were found to have an intermediate risk, and one study was labeled as high risk. Furthermore, all the included studies were found to have a low risk of bias in terms of the quality of the statistical analysis, except for two studies, which had a high risk of bias.

### 3.3. Study and Population Characteristics

The 10 studies obtained after the literature search and filtering were completed included 2113 participants who had received IRT as part of their treatment strategy. Of these patients, 877 had IDA. The 10 studies were heterogeneous in terms of study design, year of publication, number of cases and controls, mean participant age, and participant gender, as well as the countries where the studies were conducted. Table 1 shows the details of these variables for each individual study. The included studies were published between 1990 and 2022, and most (*n* = 7) were published from 2017 onward. Six studies were prospective case—control studies [15,19,31,32,41,42], two were prospective cohort studies [33,43], one was a quasi-experimental study [44], and one was a randomized clinical trial [45]. Furthermore, three studies were conducted in India [15,41,42], three in Turkey [19,32,43], and one in each of Denmark [31], Egypt [33], Pakistan [44], and Iran [45]. Regarding the participants’ gender distribution, among both the cases and the controls, 69% of the participants were female and 31% were male on average. However, three articles did not report the participants’ gender distribution [32,33,41]. The mean participant age in the case groups was 39.2 years, and it was 40.8 years in the control groups.

### 3.4. IDA and DM Parameters before IRT

This section provides details of participants’ IDA and DM parameters before they began IRT. Components of the complete blood counts (CBCs) and iron profiles demonstrated a range of positive and negative correlations, respectively, with HbA1c levels. One CBC parameter, the hematocrit level, positively correlated with the HbA1c level [19]. In contrast, the parameters which negatively correlated with HbA1c levels included hemoglobin [15,32,33,41,43], MCV [15,42,43], MCH [33], and RBC count [15,33,43]. The correlation between the RDW and HbA1c levels was indeterminate [42]. Nonetheless, some iron profile parameters positively correlated with HbA1c levels, such as TIBC [43] and serum iron level [15,42,43], while some negatively correlated, such as ferritin [15,19,32] and transferrin [15]. In a subsequent study, the correlation between transferrin and HbA1c levels was found to be insignificant [42]. Furthermore, the methods for measuring HbA1c levels varied among the studies included, with High Performance Liquid Chromatography (HPLC) being the most commonly used technique in five of the studies [15,19,42,43,45]. Other methods used were Fast Protein Liquid Chromatography (FPLC) [31], Turbidimetric Inhibition Immunoassay (TINIA) [32], and Exchange Microcolumn Chromatography (EMC) [33], while two studies did not specify the method used to measure HbA1c [41,44]. In addition, the severity of IDA has been correlated with HbA1c levels, and several studies variously concluded that as the severity increases, the HbA1c level increases [41], decreases [19,32], or insignificantly changes [31]. For instance, Pilla et al. revealed a positive correlation between the severity of IDA and HbA1c levels as they observed HbA1c levels of 5.5 and 6.1 in cases of mild to moderate and severe IDA, respectively [41]. On the other hand, Altuntaş et al. revealed that the severity of IDA was negatively correlated with HbA1c levels as they observed HbA1c levels of 5.6, 5.5, and 5 in cases of mild, moderate, and severe IDA, respectively [19]. However, the exact mechanisms behind this phenomenon are still being investigated. (for further analysis see Supplementary Table S6).

### 3.5. IRT and its Effect on HbA1c Levels

This section discusses the results of the 10 included studies which assessed the effect of IRT on HbA1c and the different IDA parameters. Nine of the studies found that IRT led to a decrease in HbA1c levels in IDA patients [15,31,32,33,41,42,43,44,45], while one study demonstrated the opposite [26]. In 1990, Gram-Hansen et al. treated 10 IDA patients with a mean HbA1c value of 4.9% with IRT. They found that the HbA1c levels had decreased after three weeks and observed that it had further reduced after nine weeks, reaching a mean of 4.6% [31]. In 2004, Coban et al. demonstrated the same outcome on a larger scale (50 patients). They treated a group of patients with IDA with daily oral ferrous sulfate (100 mg) for three months, and the mean HbA1c decreased from 7.4% to 6.2% [32]. In Medhu et al.’s study, higher doses of IRT (equivalent to 100 mg of elemental iron) were administered daily for three months, and the authors noted that there was a reduction in HbA1c from 5.51% before treatment to 5.04% after treatment [15]. Varshney et al. increased the IRT dosage to 100 mg of elemental iron twice a day and found that HbA1c reduced from 5.49% to 4.88% in 50 IDA patients [42]. In the most recently published case–control study, which was conducted in 2021, patients with IDA were stratified according to disease severity into “severe” and “mild to moderate” IDA subgroups. After treatment, the mean HbA1c level decreased from 6.1% and 5.5% to 5.1% and 4.6% in the severe IDA subgroup and mild to moderate IDA subgroup, respectively [41] (for further analysis see Supplementary Table S7).

These findings were also confirmed by a further two studies: a quasi-experimental trial and a randomized controlled trial. In 2002, El-agouza et al. followed 51 individuals treated for IDA with 325 mg/day of oral ferrous sulfate for 20 weeks and noted a reduction in the mean HbA1c level from 6.15% to 5.25% [33]. Aydin et al.’s findings reported in 2021 were consistent with El-agouza et al.’s results—they reported that treating IDA with 270 mg/day of oral ferrous sulfate for three months led to a reduction in the mean HbA1c of the IDA patients from 7.09% to 6.69% [43]. The quasi-experimental trial was undertaken by Mustafa et al., and they reported a decrease in the HbA1c level from 6.99% (pretreatment) to a mean level of 6.29% (post-treatment) after providing 182 IDA patients with oral ferrous sulfate at a dose of 200 mg/day for three months [44]. The strongest evidence was generated by Nasli-Esfahani et al., who conducted a randomized controlled trial in which the intervention group received 200 mg/day of oral iron for three months, and the control group received an oral placebo for the same period. They reported that the mean HbA1c decreased from 7.59% and 7.40% to 6.80% and 7.14% in the intervention and placebo groups, respectively [45]. Contradictory findings were reported by Altuntas et al. in 2021, who conducted a case–control study in which 131 IDA patients were administered elemental iron at a dose of 100 mg/day for three months and reported an increase in mean HbA1c values from 5.4% to 5.5% [19].

### 3.6. Meta-Regression Analysis

**Figure 2:**
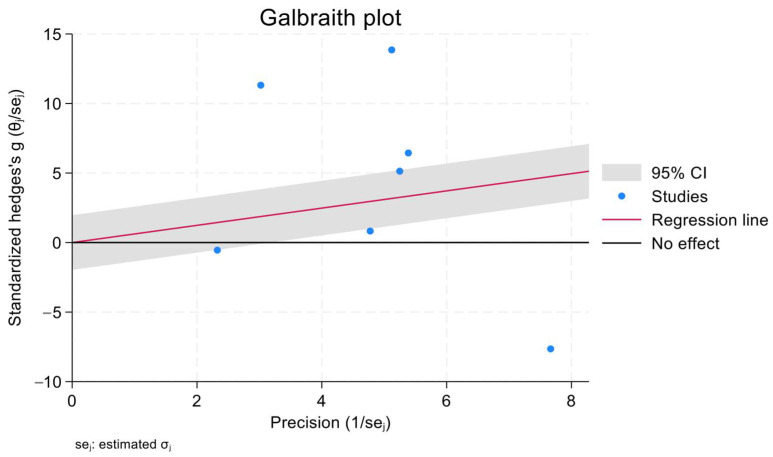
Scatter plot (Galbraith plot) which investigated the presence of heterogeneity.

**Figure 3:**
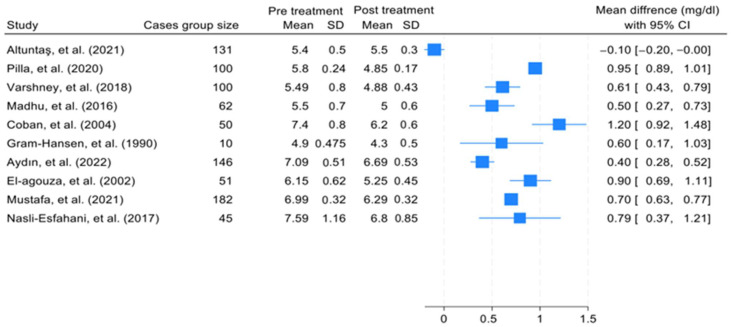
Forest plot which shows the mean difference in HbA1c levels in the cases of IDA before and after treatment [15,19,31,32,33,41,42,43,44,45].

**Figure 4:**
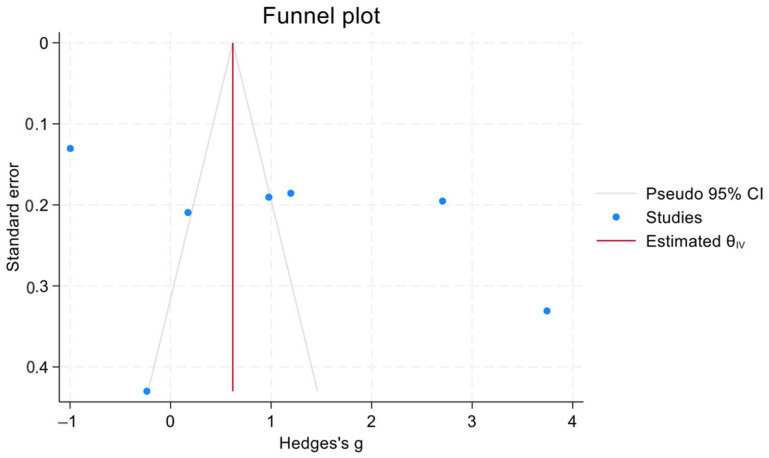
Funnel plot which investigated the presence of publication bias.

The effect size of the pooled SMD in HbA1c levels between the treatment groups and the control groups of patients with IDA was 1.8 (95% confidence interval [CI] = −0.5, 2.31). Heterogeneity was assessed using the χ^2^ and I^2^ tests, and the results were 98.46% and *p* = 0.09, respectively. In addition, a Galbraith plot was generated and showed that three of the included studies were distant from the 95% CI of the effect size (Figure 2). Moreover, (Figure 3) shows the mean difference between the HbA1c levels in cases with IDA pretreatment and post-treatment. All studies except that of Altuntas et al. showed a drop in the HbA1c level which ranged from 1.20 mg/dL to 0.43 mg/dL. The generated funnel plot shows that there was a fair distribution of the studies; however, this cannot be definitively concluded due to the limited number of included studies (Figure 4).

## 4. Discussion

The current literature depicts a spectrum of effects of IRT on HbA1c levels in diabetic and nondiabetic patients with IDA. Most of the relevant studies have used a prospective case–control design, and most of the studied cases have been middle-aged (mean age = 39.2 years) females. Nonetheless, in the majority of studies, HbA1c and IDA parameter values have been recorded pre-IRT and post-IRT to reflect any chronological correlation. In the current study, we collected and analyzed available published data to generate findings which practitioners can use to aid their decision making and to counter the notion that the HbA1c level can be used as the sole diagnostic marker for DM. The decision-making process targeted by this study involves determining which antidiabetic medications or IRT should be prescribed first for a patient with IDA and DM.

Although the HbA1c level is used as a diagnostic tool, there are several factors which must be considered prior to clinical judgment which affect the reliability of the HbA1c test. These factors range from conditions affecting erythropoiesis (e.g., IDA-associated increases in RBC half-life with HbA1c elevation), hemoglobinopathies (i.e., associated extravascular and intravascular hemolysis can have varying effects on the HbA1c content analyzed), and drugs (e.g., Ribavirin induces hemolysis and decreases HbA1c) [46,47,48,49,50]. In addition, it has been postulated that the variation in the IDA–HbA1c correlation results reported in the literature may be related to the analytical equipment and techniques used. For example, HPLC is commonly used, a technique that relies on ion concentration differences to separate hemoglobin variants. Hence, it is possible that clinical or subclinical hemoglobinopathies could interfere with HPLC-based analysis [51].

### 4.1. IDA and HbA1c (Clinical Overview and Pathophysiology)

IDA is a disorder that results in reduced iron stores and leads to an impaired capacity to produce RBCs, erythropoiesis, causing anemia [52]. IDA can be diagnosed when there is laboratory evidence of low hemoglobin, low serum iron, low serum ferritin, low transferrin saturation, and high TIBC [51]. Clinically, patients might experience fatigue, weakness, chest pain, shortness of breath, dizziness or light-headedness, cold extremities, or pica [53].

IDA and other types of anemia may affect the levels of HbA1c by altering the RBC turnover [54,55,56]. For instance, vitamin B12 deficiency anemia affects the lifespan of RBCs and decreases their turnover, eventually leading to higher HbA1c levels [41,46,57]. Conversely, in conditions associated with decreased RBC lifespan (i.e., hemolysis), such as hemoglobinopathies, splenomegaly, and rheumatoid arthritis, or the use of certain medications, it was proposed that the HbA1c level will be lower than expected, as glycated RBCs are removed from the circulation at a faster rate than normal [46,49,50,58,59,60,61,62].

The consequences of IDA are far-reaching, as RBCs constitute a significant part of many physiological processes, including DM diagnosis. In DM, monitoring the HbA1c level is integral to the diagnostic and follow-up evaluation. However, researchers have suggested that since RBCs and hemoglobin are abnormal in nature in IDA, HbA1c may also be affected [63]. It has been speculated that the IDA-induced turnover of RBCs and the changes in the hemoglobin configuration could play roles in the falsely elevated HbA1c concentrations in IDA patients [46]. However, a clear explanation of the mechanism responsible for the changes in HbA1c has yet to be provided, warranting further studies to confirm and elucidate the role of IDA.

### 4.2. IRT Effect on HbA1c

The aim of IDA management is to correct RBC production and turnover, which in turn normalizes the HbA1c level and corrects the abnormality. As reported here, several studies have indeed shown significant decreases in HbA1c in IDA cases after the administration of the appropriate therapy [15.31–33.41–45.57]. These studies used different daily doses of oral ferrous sulfate (100–350 mg) and different treatment periods (3–20 weeks) with no clear explanation as to why a specific dose or treatment period was selected. It is therefore unsurprising that the reduction in HbA1c varied among the studies. Interestingly, neither higher amounts of iron supplementation nor longer durations of therapy resulted in further improvement in HbA1c levels, implying that the relationship between iron supplementation and HbA1c may be more complex than initially thought [15,31,32,33,41,42,43,44,45]. Moreover, there was a consistent rise in hemoglobin levels across all studies, ranging from 0.7 to 5.1, after IRT. This trend could potentially serve as a future measure of the impact of IRT on HbA1c levels. It is worth noting that the study reporting the smallest increase in hemoglobin following IRT also demonstrated an increase in HbA1c levels, possibly explaining its divergence from the findings of other studies. In addition, this variance of results in this study may be attributed to the inhomogeneous distribution of the sample in their study, such as having only two severe IDA patients out of 131 participants [19].

Although this study is the first to provide a thorough review of this significant subject, and the findings could impact current practices, it has some limitations. First, clinical trial studies can have unmeasured confounders, along with the selection bias that is already acknowledged in cohort studies. Second, the studies analyzed in this systematic review exhibited significant heterogeneity in several aspects, such as the study design, sample characteristics, recruitment methods, IRT doses, and follow-up duration. Furthermore, our study is limited by the over-representation of female participants (69%) and the small sample sizes of the included studies. Therefore, the findings should be considered preliminary and cannot be applied to any particular population. Third, the included studies have used four distinct methods to measure the HbA1c level, potentially affecting the accuracy of the results. Fourth, the studies included did not establish a correlation between the root cause of IDA and HbA1c levels, leaving an unanswered question of how each cause of IDA could affect HbA1c levels and their response to IRT. Fifth, the lack of research that compares HbA1c levels with other diagnostic tests has hindered our ability to accurately assess the impact of IRT on the tests used for diagnosing DM. Finally, we must consider the limited number and scope of the studies analyzed, which may introduce a potential bias in the results of the systematic review due to potential publication and reporting biases.

## 5. Conclusions

The results discussed lead to the following clinical recommendations:

1. Clinicians should not rely only on HbA1c measurements when determining the treatment course for diabetic or nondiabetic patients with IDA, but rather should perform a comprehensive assessment.
2. HbA1c measurements should be considered when planning a diabetic patient’s glycemic control strategy, and compliance with prescribed medications must be fully encouraged.
3. It is necessary to treat IDA in diabetic patients prior to determining their daily doses of antidiabetic medications, as the presence of IDA may result in a premature elevation in the doses of these medications, which could increase the risk of drug-related complications.

However, the findings of this study are not statistically significant and are inconclusive due to the small number of studies analyzed. Consequently, there is an urgent need to conduct more studies with robust methodologies to establish the relationship between IDA, HbA1c, and IRT.

## Figures and Tables

**Table 1:** Baseline characteristics of included articles.

#	Author	Country	Study Design	Sample Size: (Control; Cases)	Median Age in Years		Female (%)
					Cases	Control	
1	Altuntaş, et al. (2021) [19]	Turkey	Prospective case—control study	(132; 131)	39 ± 10	41 ± 9	89
2	Pilla, et al. (2020) [41]	India	Prospective case—control study	(100; 100)	38	40	-
3	Varshney, et al. (2018) [42]	India	Prospective case—control study	(50; 100)	32.14 ± 3.11	35.60 ± 4.57	54
4	Madhu, et al. (2016) [15]	India	Prospective case—control study	(60; 62)	31.4 ± 11.3	32.5 ± 10.1	Case = 87.1 Control = 85
5	Coban, et al. (2004) [32]	Turkey	Prospective case—control study	(50; 50)	35.7 ± 11.9		60
6	Gram-Hansen, et al. (1990) [31]	Denmark	Prospective case—control study	(10; 10)	52 ± 13.5	60 ± 5.5	-
7	Aydın, et al. (2022) [43]	Turkey	Prospective cohort study	(0; 146)	45 ± 2.5		82.1
8	El-agouza, et al. (2002) [33]	Egypt	Prospective cohort study	(0; 51)	University school age		-
9	Mustafa, et al. (2021) [44]	Pakistan	Quasi-experimental trial	(0; 182)	54.84 ± 8.69		45
10	Nasli-Esfahani, et al. (2017) [45]	Iran	Randomized clinical trial	(45; 45)	51.47 ± 7.07	52 ± 7.35	70

## Data Availability

Not applicable.

## Supplementary Materials

The following supporting information can be downloaded at: https://www.mdpi.com/article/10.3390/jcm12237287/s1, Table S1: Keywords used to screen for eligible articles; Table S2: Joanna Briggs Institute (JBI) critical appraisal checklists for assessing the quality of studies; Table S3: The results of the different JBI questionnaires (questionnaire for randomized controlled trials, cohort studies, case—control studies and quasi-experimental studies); Table S4: Keywords used to screen for eligible articles; Table S5: A detailed evaluation of the risk of bias of all the included studies; Table S6: Baseline characteristics of the IDA and DM parameters; Table S7: The difference in IDA parameters and HbA1c levels after using the iron replacement therapy.

## References

[1] Andrews N.C. (1999). The iron transporter DMT1. Int. J. Biochem. Cell Biol..

[2] Adams P.C., Reboussin D.M., Barton J.C., McLaren C.E., Eckfeldt J.H., McLaren G.D., Dawkins F.W., Acton R.T., Harris E.L., Gordeuk V.R. (2005). Hemochromatosis and iron-overload screening in a racially diverse population. N. Engl. J. Med..

[3] Salonen J.T., Tuomainen T.-P., Nyyssönen K., Lakka H.-M., Punnonen K. (1998). Relation between iron stores and non-insulin dependent diabetes in men: Case-control study. BMJ.

[4] U.S. National Library of Medicine https://pubmed.ncbi.nlm.nih.gov/31082013/.

[5] Abbaspour N., Hurrell R., Kelishadi R. (2014). Review on iron and its importance for human health. J. Res. Med. Sci..

[6] Liu J., Li Q., Yang Y., Ma L. (2020). Iron metabolism and type 2 diabetes mellitus: A meta-analysis and systematic review. J. Diabetes Investig..

[7] World Health Organization Use of Glycated Haemoglobin (HbA1c) in Diagnosis of Diabetes Mellitus. https://www.who.int/publications-detail-redirect/use-of-glycated-haemoglobin-(-hba1c)-in-diagnosis-of-diabetes-mellitus.

[8] Massi-Benedetti M. (2006). Changing targets in the treatment of type 2 diabetes. Curr. Med. Res. Opin..

[9] The International Expert Committee (2009). International Expert Committee Report on the role of the A1C assay in the diagnosis of diabetes. Diabetes Care.

[10] American Diabetes Association (2010). Diagnosis and classification of diabetes mellitus. Diabetes Care.

[11] Horton B.F., Huisman T.H. (1965). Studies on the heterogeneity of haemoglobin. VII. Minor haemoglobin components in haematological diseases. Br. J. Haematol..

[12] Christy A.L., Manjrekar P.A., Babu R.P., Hegde A., Rumkini M.S. (2014). Influence of Iron Deficiency Anemia on Hemoglobin A1C Levels in Diabetic Individuals with Controlled Plasma Glucose Levels. Iran Biomed. J..

[13] Prasad K., Gupte S. (2020). Correlation of Iron Deficiency Anemia on HbA1C Levels: Comparison among Patients living with Diabetic and Non-diabetics. Int. J. Health Clin. Res..

[14] Chaudhari A.S., Sontakke A.N., Trimbake S.B. (2020). HbA1c status in Type II Diabetes Mellitus with and without Iron Deficiency Anemia. Int. J. Biochem. Res. Rev..

[15] Madhu S.V., Raj A., Gupta S., Giri S., Rusia U. (2017). Effect of iron deficiency anemia and iron supplementation on HbA1c levels—Implications for diagnosis of Prediabetes and diabetes mellitus in Asian Indians. Clin. Chim. Acta.

[16] Urrechaga E. (2018). Influence of iron deficiency on Hb A1C levels in type 2 diabetic patients. Diabetes Metab. Syndr..

[17] Hong J.W., Ku C.R., Noh J.H., Ko K.S., Rhee B.D., Kim D.-J. (2015). Association between the presence of iron deficiency anemia and hemoglobin A1C in Korean adults. Medicine.

[18] Sharma A., Kalra H.S., Arora R., Kukreja S. (2020). Is HbA1c a reliable diagnostic marker for diabetes mellitus in patients with iron deficiency anaemia?: A Cross Sectional Study. Int. J. Clin. Biochem. Res..

[19] Altuntaş S.Ç., Evran M., Gürkan E., Sert M., Tetiker T. (2021). HbA1c level decreases in iron deficiency anemia. Wien Klin Wochenschr..

[20] Puri S.S., Gautam S., Singhal P. (2021). Correlation of iron deficiency anaemia with haemoglobin A1C at a Tertiary Care Teaching Hospital In Uttar Prades. Int. J. Health Clin. Res..

[21] Kalairajan S., K V.D., R M.A. (2019). A study on influence of iron deficiency anaemia over HbA1c levels. Int. J. Adv. Med..

[22] Solomon A., Hussein M., Negash M., Ahmed A., Bekele F., Kahase D. (2019). Effect of iron deficiency anemia on HbA1c in diabetic patients at Tikur Anbessa Specialized Teaching Hospital, Addis Ababa Ethiopia. BMC Hematol..

[23] Oğuz E., Ercan M., Yilmaz F. (2014). Effect of Iron Deficiency Anemia on Hemoglobin A1c Levels in Normoglisemic Individuals. Ank. Med. J..

[24] Mitchell T.R., Anderson D., Shepperd J. (1980). Iron deficiency, haemochromatosis, and glycosylated haemoglobin. Lancet.

[25] Silva J.F., Pimentel A.L., Camargo J.L. (2016). Effect of iron deficiency anaemia on HbA1c levels is dependent on the degree of anaemia. Clin. Biochem..

[26] Rajagopal L., Arunachalam S., Ganapathy S., Ramraj B., Raja V. (2017). A comparison of effect of Iron Deficiency Anemia on HbA1c levels in controlled diabetics and non-diabetics: A cross sectional analysis of 300 cases. Ann. Pathol. Lab. Med..

[27] Bansal R.K., Yadav Y.R., Kulkarni H.S., Garg S., Jain P., Sharma V.K., Maheshwari S. (2020). Effect of Iron Deficiency Anemia on HbA1c in Non-Diabetics. J. Diabetes Endocrinol. Assoc. Nepal.

[28] Rajagopal L., Ganapathy S., Arunachalam S., Raja V., Ramraj B. (2017). Does Iron Deficiency Anaemia and its Severity Influence HbA1C Level in Non Diabetics? An Analysis of 150 Cases. J. Clin. Diagn. Res..

[29] Jimenez K., Kulnigg-Dabsch S., Gasche C. (2015). Management of Iron Deficiency Anemia. Gastroenterol. Hepatol..

[30] Brooks A.P., Metcalfe J., Day J.L., Edwards M.S. (1980). Iron deficiency and glycosylated haemoglobin A. Lancet.

[31] Gram-Hansen P., Eriksen J., Mourits-Andersen T., Olesen L. (1990). Glycosylated haemoglobin (HbA1c) in iron- and vitamin B12 deficiency. J. Intern. Med..

[32] Coban E., Ozdogan M., Timuragaoglu A. (2004). Effect of iron deficiency anemia on the levels of hemoglobin A1C in nondiabetic patients. Acta Haematol..

[33] El-Agouza I., Abu Shahla A., Sirdah M. (2002). The effect of iron deficiency anaemia on the levels of haemoglobin subtypes: Possible consequences for clinical diagnosis. Clin. Lab. Haematol..

[34] Hashimoto K., Noguchi S., Morimoto Y., Hamada S., Wasada K., Imai S., Murata Y., Kasayama S., Koga M. (2008). A1C but Not Serum Glycated Albumin Is Elevated in Late Pregnancy Owing to Iron Deficiency. Diabetes Care.

[35] Hashimoto K., Koga M. (2015). Indicators of glycemic control in patients with gestational diabetes mellitus and pregnant women with diabetes mellitus. World J. Diabetes.

[36] Tarim Ö., Küçükerdogan A., Günay Ü., Eralp Ö., Ercan İ. (1999). Effects of iron deficiency anemia on hemoglobin A1C in type 1 diabetes mellitus. Pediatr. Int..

[37] Sinha N., Mishra T.K., Singh T., Gupta N. (2012). Effect of iron deficiency anemia on hemoglobin A1C levels. Ann. Lab. Med..

[38] AlQarni A., Alghamdi A., Aljubran H., Bamalan O. The Effect of Iron Deficiency Anemia on Glycated Hemoglobin A1c: A Systematic Review and Meta-Analysis, PROSPERO 2022 CRD42022312203. https://www.crd.york.ac.uk/prospero/display_record.php?ID=CRD42022312203.

[39] Tufanaru C., Munn Z., Aromataris E., Campbell J., Hopp L. (2020). Chapter 3: Systematic reviews of effectiveness. JBI Manual for Evidence Synthesis.

[40] Moola S., Munn Z., Tufanaru C., Aromataris E., Sears K., Sfetcu R., Currie M., Lisy K., Qureshi R., Mattis P., Aromataris E., Munn Z. (2020). Chapter 7: Systematic reviews of etiology and risk. JBI Manual for Evidence Synthesis.

[41] Pilla R., Palleti S.K., Rayala R., Skss S.R., Abdul Razzack A., Kalla S. (2020). Glycated Haemoglobin (HbA1c) Variations in Nondiabetics With Nutritional Anemia. Cureus.

[42] Varshney A.K., Singhal S., Gupta P.K., Taneja R.S., Chawla M.P.S., Tonk R.S., Mahto S.K., Sharma L.K. (2018). Effect of Iron Supplementation on Glycosylated Haemoglobin in Non-Diabetic Individuals with Iron Deficiency Anaemia. J. Indian Acad. Clin. Med..

[43] Aydın B., Özçelik S., Kilit T.P., Eraslan S., Çelik M., Onbaşı K. (2022). Relationship between glycosylated hemoglobin and iron deficiency anemia: A common but overlooked problem. Prim. Care Diabetes.

[44] Mustafa R., Uthman M. (2021). Effect of Iron Replacement on Mean Decrease of Hba1c in Diabetic Type 2 Patients with Iron Deficiency Anemia. Pak. J. Med. Health Sci..

[45] Nasli-Esfahani E., Larijani B., Amini P., Ghodssi-Ghassemabadi R., Razmandeh R. (2017). Effect of treatment of iron deficiency anemia on hemoglobin A1C in type 2 diabetic patients. Turk. J. Med. Sci..

[46] Gallagher E.J., Roith D.L., Bloomgaren Z. (2009). Review of hemoglobin A1c in the management of diabetes. J. Diabetes.

[47] Kesson C.M., Whitelaw J.W., Ireland J.T. (1979). Drug-induced haemolysis and fast haemoglobin A1 in diabetes mellitus. Br. Med. J..

[48] Tack C.J., Wetzels J.F. (1996). Decreased HbA1c levels due to sulfonamide-induced hemolysis in two IDDM patients. Diabetes Care.

[49] Robertson M. (2008). Artificially low HbA1c associated with treatment with ribavirin. BMJ.

[50] Diop M.-E., Bastard J.-P., Meunier N., Thévenet S., Maachi M., Capeau J., Pialoux G., Vigouroux C. (2006). Inappropriately low glycated hemoglobin values and hemolysis in HIV-infected patients. AIDS Res. Hum. Retroviruses.

[51] Yedla N., Kuchay M.S., Mithal A. (2015). Hemoglobin E disease and glycosylated hemoglobin. Indian J. Endocrinol. Metab..

[52] Johnson-Wimbley T.D., Graham D.Y. (2011). Diagnosis and management of iron deficiency anemia in the 21st century. Ther. Adv. Gastroenterol..

[53] Kumar A., Sharma E., Marley A., Samaan M.A., Brookes M.J. (2022). Iron deficiency anaemia: Pathophysiology, assessment, practical management. BMJ Open Gastroenterol..

[54] Little R.R., Sacks D.B. (2009). HbA1c: How do we measure it and what does it mean?. Curr. Opin. Endocrinol. Diabetes Obes..

[55] World Health Organization. https://www.who.int/health-topics/anaemia.

[56] Shepard J.G., Airee A., Dake A.W., McFarland M.S., Vora A. (2015). Limitations of A1c Interpretation. South Med. J..

[57] Maheshwari V.D., Capoor S., Chaturvedi S., Manglunia A., Singla A. (2017). Impact of Iron and Vitamin B12 Anaemia at Glycosylated Hemoglobin Level: A Case Control Study. IOSR J. Dent. Med. Sci..

[58] Bry L., Chen P.C., Sacks D.B. (2001). Effects of Hemoglobin Variants and Chemically Modified Derivatives on Assays for Glycohemoglobin. Clin. Chem..

[59] Albright E.S., Ovalle F., Bell D.S.H. (2002). Artificially low hemoglobin A1c caused by use of dapsone. Endocr. Pract..

[60] Bernstein R.M., Freedman D.B., Liyanage S.P., Dandona P. (1982). Glycosylated haemoglobin in rheumatoid arthritis. Ann. Rheum. Dis..

[61] Weykamp C. (2013). HbA1c: A Review of Analytical and Clinical Aspects. Ann. Lab. Med..

[62] Hellman R. (2016). When are HBA1C Values Misleading?. AACE Clin. Case Rep..

[63] ElSayed N.A., Aleppo G., Aroda V.R., Bannuru R.R., Brown F.M., Bruemmer D., Collins B.S., Hilliard M.E., Isaacs D., Johnson E.L. (2023). 2. classification and diagnosis of diabetes: Standards of care in Diabetes-2023. Diabetes Care.

