# Supplementary material for: The Effect of Iron Replacement Therapy on HbA1c Levels in Diabetic and Nondiabetic Patients: A Systematic Review and Meta-Analysis
Source: J Clin Med. 2023 Nov 24;12(23):7287. doi: 10.3390/jcm12237287 (PMC10707099; doi:10.3390/jcm12237287)
Supplement: Supplementary file 1 [file jcm-12-07287-s001.zip › jcm-2664347-supplementary.pdf]

Supplementary Table S1. Keywords used to screen for eligible articles.

#	Search strategy steps in Medline database	Search strategy keywords in Medline database
**1**	Iron deficiency keywords	Iron deficiency anemia OR Iron deficiency anaemia OR Iron deficient OR Iron deficit OR Iron insufficient OR Iron insufficiency OR Total iron binding capacity OR Ferritin OR serum Iron OR Iron therapy OR Iron supplementation
**2**	HbA1c keywords	HbA1c OR glycated hemoglobin A1c OR glycated haemoglobin A1c OR glycosylated Haemoglobin OR glycosylated hemoglobin OR hemoglobin A1c OR Haemoglobin A1c OR glycohemoglobin OR diabetes OR prediabetes
**3**	Merged	1 AND 2
#	**Search strategy steps in Embase database**	**Search strategy keywords in Embase database**
**1**	Iron deficiency keywords	Iron deficiency anemia OR Iron deficiency anaemia OR Iron deficient OR Iron deficit OR Iron insufficient OR Iron insufficiency OR Total iron binding capacity OR Ferritin OR serum Iron OR Iron therapy OR Iron supplementation
**2**	HbA1c keywords	HbA1c OR glycated hemoglobin A1c OR glycated haemoglobin A1c OR glycosylated Haemoglobin OR glycosylated hemoglobin OR hemoglobin A1c OR Haemoglobin A1c OR glycohemoglobin OR diabetes OR prediabetes
**3**	Merged	1 AND 2
#	**Search strategy steps in Cochrane database**	**Search strategy keywords in Cochrane database**
**1**	Iron deficiency keywords	Iron deficiency anemia OR Iron deficiency anaemia OR Iron deficient OR Iron deficit OR Iron insufficient OR Iron insufficiency OR Total iron binding capacity OR Ferritin OR serum Iron OR Iron therapy OR Iron supplementation
**2**	HbA1c keywords	HbA1c OR glycated hemoglobin A1c OR glycated haemoglobin A1c OR glycosylated Haemoglobin OR glycosylated hemoglobin OR hemoglobin A1c OR Haemoglobin A1c OR glycohemoglobin OR diabetes OR prediabetes
**3**	Merged	1 AND 2

Supplementary Table S2. Joanna Briggs Institute (JBI) critical appraisal checklists for assessing the quality of studies.

RCT	Question	Low risk of bias	Intermediate risk of bias	High risk of bias
Information bias	4,5,6,10,11	Answer Yes 4/5 times	Answer Yes 3 times	Answer Yes 0/1/2 times
Selection bias	1,2,3,7,8,9	Answer Yes 5/6 times	Answer Yes 3/4 times	Answer Yes 0/1/2 times
Confounding	-	-	-	-
Statistical quality	12,13	Answer Yes 2 times	Answer Yes 1 times	Answer Yes 0 times

Cohort	Question	Low risk of bias	Intermediate risk of bias	High risk of bias
Information bias	2,3,7,8,9,10	Answer Yes 5/6 times	Answer Yes 3/4 times	Answer Yes 0/1/2 times
Selection bias	1,6	Answer Yes 2 times	Answer Yes 1 times	Answer Yes 0 times
Confounding	4,5	Answer Yes 2 times	Answer Yes 1 times	Answer Yes 0 times
Statistical quality	11	Answer Yes 1 times		Answer Yes 0 times

Case control	Question	Low risk of bias	Intermediate risk of bias	High risk of bias
Information bias	4,5,8,9	Answer Yes 4 times	Answer Yes 2/3 times	Answer Yes 0/1 times
Selection bias	1,2,3	Answer Yes 3 times	Answer Yes 1/2 times	Answer Yes 0 times
Confounding	6,7	Answer Yes 2 times	Answer Yes 1 times	Answer Yes 0 times
Statistical quality	10	Answer Yes 1 times	-	Answer Yes 0 times

Quasi-experimental	Question	Low risk of bias	Intermediate risk of bias	High risk of bias
Information bias	4,5,6,7	Answer Yes 4 times	Answer Yes 2/3 times	Answer Yes 0/1 times
Selection bias	1,2,3,8	Answer Yes 4 times	Answer Yes 2/3 times	Answer Yes 0/1 times
Confounding	-	-	-	-
Statistical quality	9	Answer Yes 1 times	-	Answer Yes 0 times

Supplementary Table. S3: The results of the different JBI questionnaires (questionnaire for randomized controlled trial, cohort studies, case control studies and quasi-experimental studies).

**RCT**	**Q1**	**Q2**	**Q3**	**Q4**	**Q5**	**Q6**	**Q7**	**Q8**	**Q9**	**Q10**	**Q11**	**Q12**	**Q13**
Naslı-Esfahani	Yes	Yes	Yes	Yes	No	Yes	No	Yes	Yes	Yes	Yes	Yes	Yes
**Cohort**	**Q1**	**Q2**	**Q3**	**Q4**	**Q5**	**Q6**	**Q7**	**Q8**	**Q9**	**Q10**	**Q11**		
Aydın	NA	NA	Yes	Yes	Yes	NA	Yes	Yes	Yes	UC	Yes		
El-Agouza	NA	NA	Yes	Yes	UC	NA	Yes	Yes	Yes	UC	UC		
**Case control**	**Q1**	**Q2**	**Q3**	**Q4**	**Q5**	**Q6**	**Q7**	**Q8**	**Q9**	**Q10**			
Altuntaş	Yes	Yes	Yes	Yes	Yes	Yes	Yes	Yes	Yes	Yes			
Pilla	No	Yes	Yes	Yes	Yes	No	Yes	Yes	Yes	Yes			
Varshney	Yes	Yes	Yes	Yes	Yes	Yes	Yes	Yes	Yes	Yes			
Madhu	Yes	No	Yes	Yes	Yes	No	Yes	Yes	Yes	Yes			
Coban	Yes	Yes	Yes	Yes	Yes	No	Yes	Yes	Yes	Yes			
Gram-Hansen	No	No	Yes	Yes	Yes	No	No	Yes	Yes	Yes			
**Quasi-experimental**	**Q1**	**Q2**	**Q3**	**Q4**	**Q5**	**Q6**	**Q7**	**Q8**	**Q9**				
Mustafa	Yes	Yes	No	No	Yes	UC	Yes	Yes	UC				

Supplementary Table S4. Keywords used to screen for eligible articles.

#	Search strategy in Medline database	Results
**1**	Iron deficiency anemia OR Iron deficiency anaemia OR Iron deficient OR Iron deficit OR Iron insufficient OR Iron insufficiency OR Total iron binding capacity OR Ferritin OR serum Iron OR Iron therapy OR Iron supplementation	N = 102,825
**2**	HbA1c OR glycated hemoglobin A1c OR glycated haemoglobin A1c OR glycosylated Haemoglobin OR glycosylated hemoglobin OR hemoglobin A1c OR Haemoglobin A1c OR glycohemoglobin OR diabetes OR prediabetes	N = 1,014,962
**3**	1 AND 2	N = 6,383
**#**	**Search strategy in Embase database**	**Results**
**1**	Iron deficiency anemia OR Iron deficiency anaemia OR Iron deficient OR Iron deficit OR Iron insufficient OR Iron insufficiency OR Total iron binding capacity OR Ferritin OR serum Iron OR Iron therapy OR Iron supplementation	N = 26,855
**2**	HbA1c OR glycated hemoglobin A1c OR glycated haemoglobin A1c OR glycosylated Haemoglobin OR glycosylated hemoglobin OR hemoglobin A1c OR Haemoglobin A1c OR glycohemoglobin OR diabetes OR prediabetes	N = 1,423,164
**3**	1 AND 2	
**#**	**Search strategy in Cochrane database**	**Results**
**1**	Iron deficiency anemia OR Iron deficiency anaemia OR Iron deficient OR Iron deficit OR Iron insufficient OR Iron insufficiency OR Total iron binding capacity OR Ferritin OR serum Iron OR Iron therapy OR Iron supplementation	N = 274
**2**	HbA1c OR glycated hemoglobin A1c OR glycated haemoglobin A1c OR glycosylated Haemoglobin OR glycosylated hemoglobin OR hemoglobin A1c OR Haemoglobin A1c OR glycohemoglobin OR diabetes OR prediabetes	N = 2625
**3**	1 AND 2	N = 264

Author	Information Bias	Selection Bias	Confounding	Statistical Quality
Altuntaş, et al. (2021)	+	+	+	+
Pilla, et al. (2020)	+	+/-	+/-	+
Varshney, et al. (2018)	+	+	+	+
Madhu, et al. (2016)	+	+/-	+/-	+
Coban, et al. (2004)	+	+	+/-	+
Gram-Hansen, et al. (1990)	+	+/-	-	+
Aydın, et al. (2022)	+/-	-	+	+
El-agouza, et al. (2002)	+/-	-	+/-	-
Mustafa, et al. (2021)	+/-	+/-	NA	-
Nasli-Esfahani, et al. (2017)	+	+	NA	+

Supplementary Table S5: A detailed evaluation of the risk of bias of all the included studies.

Abbreviations: (+) Low risk of bias, (+/-) intermediate risk of bias, (-) high risk of bias, (NA) not applicable.

Supplementary Table S6. Baseline characteristics of the IDA and DM parameters.

Author	IDA																						DM and HbA1c								
	Hemoglobin		Ferritin		Transferrin		Iron level		TIBC		MCV		MCH		MCHC		RBC count		Hematocrit		RDW		DM in cases	DM in control	Messured HbA1c	HbA1c level		FBG		Postprandial blood sugar	
	Cases	Control	Cases	Control	Cases	Control	Cases	Control	Cases	Control	Cases	Control	Cases	Control	Cases	Control	Cases	Control	Cases	Control	Cases	Control				Cases	Control	Cases	Control	Cases	Control
Altuntaş, et al. (2021)	11.545 ± 1.6*	13.1 ± 1.3	6.6 ± 5.03*	40 ± 52	-	-	-	-	-	-	79 ± 10*	87 ± 4	-	-	-	-	-	-	36.8 ± 4.64*	39 ± 3.5	-	-	0	0	HPLC	5.4 ± 0.5*	5.9 ± 0.5	-	-	-	-
Pilla, et al. (2020)	7.8 ± 0.46*	-	-	-	-	-	-	-	-	-	-	-	-	-	-	-	-	-	-	-	-		0	0	-	5.8 ± 0.24 *	5.2 ± 0.2	-	-	-	-
Varshney, et al. (2018)	9.73 ± 0.83	14.00 ± 0.72	7.50 ± 2.38	131.91 ± 17.88	10 ± 2.1	40.63 ± 4.34	34.24 ± 4.98	123.38 ± 20.13	552.5 ± 43.12	304.12 ± 45.78	70.9 ± 9.27	94.2 ± 6.61	22.2 ± 3.73	31.74 ± 3.22	30.4 ± 3.38	34.28 ± 2.44	3.85 ± 0.48	4.98 ± 0.37	-	-	9.1 ± 1.62	= 13.4 ± 1.9	0	0	HPLC	5.49 ± 0.8	4.68 ± 0.28	86.22 ± 10.72	84.52 ± 8.74	136.28 ± 16.24	130.68 ± 10.10
Madhu, et al. (2016)	7.4 ± 1.2*	13.4 ± 1.3	17.3 ± 3*	315.9 ± 63.8	8.1 ± 2.2*	28.7 ± 8.2	5.4 ± 1.4*	17.3 ± 4.5	73.3 ± 1.7*	57.9 ± 6.8	66.7 ± 7.3*	89.7 ± 9.7	20.1 ± 3.9*	30.1 ± 2.9	29.3 ± 2.1*	32.6 ± 1.3	3.7 ± 0.7*	4.6 ± 0.6	24.9 ± 3.5*	41.3 ± 4.7	-	-	6 diabetic 20 prediabetic	0	HPLC	5.5 ± 0.7	4.9 ± 0.5	-	-	-	-
Coban , et al. (2004)	10.8 ± 1.2*	13.6 ± 0.9	3.68 ± 1.78*	22.7 ± 6.3	-	-	--	-	-	-	72.2 ± 4.9*	84.4 ± 4.8	22.8 ± 2.2*	32.9 ± 1.9	-	-	-	-	33.8 ± 3*	39.9 ± 2.4	-	-	0	0	TINIA	7.4 ± 0.8*	5.2 ± 0.2	91.4 ± 9.8	89.7 ± 10.3	102.6 ± 5.8	101.7 ± 6.1
Gram-Hansen, et al. (1990)	5.1 ± 0.875 *	-	-	-	-	-	-	-	-	-	66 ± 6.25*	-	-	-	-	-	-	-	-	-	-	-	0	0	FPLC	4.9 ± 0.475*	5 ± 0.325	-	-	-	-
Aydın , et al. (2022)	10.4 ± 0.4*		4 ± 0.75*		-		20 ± 13.3*		407.78 ± 52.41*		74± 1.55*		-		-		4.4 ± 0.125*		31.95 ± 1.1125*		-		146		HPLC	7.09 ± 0.51*		118 ± 6*		-	
El-agouza, et al. (2002)	10.96 ± 1.12*		3.45 ± 1.73*		-		-		-		72.38 ± 5.15*		23.35 ± 2.23*		32.17 ± 0.87*		4.71 ± 0.35*		34.02 ± 2.98*		17.74 ± 2.13*		0		EMC	6.15 ± 0.62*		-		-	
Mustafa, et al. (2021)	-		-		-		-		-		-		-		-		-		-		-		182		-	6.99 ± 0.32		-		-	
Nasli-Esfahani, et al. (2017)	11.52 ± 0.86	11.3 ± 0.73	32.33 ± 39.23	39.37 ± 42.46	-	-	45.46 ± 29.77	47.35 ± 36.74	227.59 ± 116.27	238.37 ± 108.01	79.94 ± 6.04	82.08 ± 6.7	26.98 ± 3.27	27.65 ± 3.02	31.84 ± 1.9	31.81 ± 1.73	4.7 ± 0.55*	4.32 ± 0.42	-	-	-	-	45	45	HPLC	7.59 ± 1.16	7.4 ± 1.01	-	-	-	-

* P value <0.05 (statistically significant). HPLC: High Performance Liquid Chromatography, TINIA: Turbidimetric Inhibition Immunoassay, FPLC: Fast Protein Liquid Chromatography, EMC: Exchange Microcolumn Chromatography

Supplementary Table S7. The difference of IDA parameters and HbA1c level after using the iron replacement therapy.

Author	Number of diabetic patients in cases	Number of diabetic patients in control	Number of cases	Parameters After Iron Replacement Therapy																IRT/ HbA1c end correlation
				Oral ferrous sulfate mg/day	Duration (by weeks)	HbA1c	Fasting blood glucose	Postprandial blood sugar	Hemoglobin	Ferritin	Transferrin	Iron level	TIBC	MCV	MCH	MCHC	RBC count	Hematocrit	RDW	
Altuntaş, et al. (2021)	0	0	131	100	12	5.5 ± 0.3	90 ± 8.6*	-	12.2 ± 1.6*	15.1 ± 5*	-	-	-	82 ± 7.4*	-	-	-	38 ± 4.9*	-	Increase
Pilla, et al. (2020)	0	0	100	Not mentioned	12	4.85 ± 0.17*	-	-	12.9 ± 0.205*	-	-	-	-	-	-	-	-	-	-	Decrease
Varshney, et al. (2018)	0	0	100	Not mentioned	8	4.88 ± 0.43*	84.3 ± 8.69	134.21 ± 14.71	12.98 ± 4.01*	108 ± 12.24*	34.24 ± 4.98*	111.5 ± 12.86*	336.28 ± 56.42*	88.86 ± 6.82*	30.2 ± 4.16*	33.2 ± 3.76*	4.56 ± 0.36*	-	11.4 ± 1.31	Decrease
Madhu, et al. (2016)	6 diabetic<br>20 prediabetic	0	62	100	12	5.0 ± 0.6*	-	-	8.9 ± 1.2*	36.6 ± 10.6*	14.6 ± 2.6*	10.3 ± 2.7*	64.4 ± 2.9*	74.3 ± 8.7*	22.1 ± 3.3*	29.7 ± 1.9	4.1 ± 0.6*	29.9 ± 3.1*	-	Decrease
Coban, et al. (2004)	0	0	50	100	12	6.2 ± 0.6*	-	-	12.7 ± 0.96*	15.7 ± 1.88*	-	-	-	82.2 ± 5.1*	31.6 ± 2.1*	-	-	39.9 ± 2.4*	-	Decrease
Gram-Hansen, et al. (1990)	0	0	10	Not mentioned	3	4.3 ± 0.5*	-	-	7.2 ± 2.1*	-	-	-	-	79 ± 5.5*	-	-	-	-	-	Decrease
Aydın, et al. (2022)	146		146	270	12	6.69 ± 0.53*	116± 4.75	-	12.6 ± 0.275*	15 ± 3.05*	-	57.5 ± 8.5*	324.21 ± 67.86*	82 ± 1.5*	-	-	4.8 ± 0.5*	40.25 ± 0.55*	–	Decrease
El-agouza, et al. (2002)	0		51	325	20	5.25 ± 0.45*	-	-	13.20 ± 0.65*	28.77 ± 14.13*	-	-	-	81.30 ± 5.22*	25.52 ± 1.70*	31.42 ± 1.48*	5.19 ± 0.28*	42.08 ± 2.47*	19.58 ± 2.98*	Decrease
Mustafa, et al. (2021)	182		182	200	12	6.29 ± 0.32*	-	-	-	-	-	-	-	-	-	-	-	-	-	Decrease
Nasli-Esfahani, et al. (2017)	45	45	45	200	12	6.8 ± 0.85	131.76 ± 41.75	-	13.71 ± 1.37*	64.1 ± 56.82*	-	73.26 ± 34.27*	-	84.62 ± 4.37	28.66 ± 2.66*	33.38 ± 1.41	4.86 ± 0.41*	-	-	Decrease

* P value <0.05 (statistically significant). HPLC: High Performance Liquid Chromatography, TINIA: Turbidimetric Inhibition Immunoassay, FPLC: Fast Protein Liquid Chromatography, EMC: Exchange Microcolumn Chromatography
